# Physiotherapy Approaches for Temporomandibular Disorders: A Multimodal Conservative Management Strategy

**DOI:** 10.7759/cureus.88885

**Published:** 2025-07-28

**Authors:** Nezha Ben El Hammi, Fatimazahra Amessegher, Sofia Moudni, El Mehdi Jouhadi

**Affiliations:** 1 Department of Fixed Prosthodontics, Faculty of Dentistry, Hassan II University, Casablanca, MAR; 2 Faculty of Dentistry, Hassan II University, Casablanca, MAR

**Keywords:** conservative modalities, multidisciplinary approach, physical therapy, self-rehabilitation, temporomandibular joint disorders

## Abstract

Temporomandibular disorder (TMD) is a complex, multifactorial disorder affecting the masticatory muscles, the temporomandibular joint, and associated structures. These disorders are also associated with other symptoms affecting the head and neck region, such as headaches, ear-related symptoms, cervical spine dysfunction, and altered head and neck posture. TMDs require multidisciplinary care. Physiotherapy is one of the pillars of TMD management; it is part of the non-invasive treatment recommended by medical guidelines. It aims to eliminate or significantly reduce pain and functional discomfort, reduce muscle tone, and increase the range of mandibular movement. Physiotherapy also remains a preferred therapeutic choice in the management of generalized myalgia, tension headaches, and myofascial pain. Physiotherapy modalities include behavioral education, physical therapy, manual therapy, and self-rehabilitation. This article aims to determine the major interest that physiotherapy brings to the treatment of TMDs while detailing these modalities.

## Introduction and background

Temporomandibular disorders (TMDs) encompass a wide range of clinical conditions that affect the masticatory muscles, the temporomandibular joint (TMJ), and the adjacent bone and soft tissue structure either independently or in combination [1].

The global prevalence of TMDs is estimated at 34%, with significant variations according to geographic region and age group. South America has the highest prevalence (47%), followed by Asia (33%) and Europe (29%). Adults aged 18 to 60 years make up the most affected population (41%), with a clear predominance of women. These data underline the importance of TMDs as a global public health issue [2].

These disorders are typically characterized by the presence of at least one of three cardinal clinical signs [3]: audible noises (clicking, popping, or grating) during mandibular movements, the reproducibility of which is an indicator of dysfunction; facial pain, intermittent or persistent, often modulated by movement or palpation of the masticatory muscles and/or TMJ; or restricted range of motion, deviations or deflections of the mandible, revealing a mechanical alteration.

Painful TMDs result from a multifactorial interaction combining psychological vulnerability (stress, anxiety, depression), somatic factors (functional syndromes, locoregional symptoms), and genetic, mechanical (bruxism, microtrauma), and environmental influences (chronic stress, postures). This pathophysiological complexity, characteristic of chronic pain, justifies comprehensive management, integrating biological, psychological, and social dimensions [4].

The management of TMDs should be rational, individualized, non-invasive, and multimodal [5,6].

Treatment goals include reassuring the patient, reducing or eliminating pain, enhancing muscle function, improving mandibular range of motion, and enhancing overall functional comfort.

The management of TMDs encompasses four primary therapeutic approaches [3,6]: conservative treatments (e.g., physical therapy, pharmacotherapy, and injections), orthopedic interventions (e.g., occlusal splints), structural therapies (e.g., prosthetic rehabilitation, orthodontics, and maxillofacial surgery), and preventive/interceptive measures.

In this article, we focus on the role of physiotherapy as a core component of conservative treatment in the comprehensive management of temporomandibular dysfunction.

## Review

Physiotherapy in the treatment of TMDs

Physiotherapy primarily aims to restore or maintain optimal physical function. It is highly advantageous for patients suffering from chronic musculoskeletal conditions, which are often associated with pain, reduced range of motion, impaired muscle function, or decreased physical fitness [7].

Physiotherapeutic interventions are tailored to the patient’s clinical profile and may be passive (applied by the therapist), active (performed by the patient under supervision), or self-directed (integrated into daily life via home programs). They encompass a range of techniques, including patient education, physical modalities, manual therapy, exercise therapy, and self-rehabilitation [1,8-10].

Education

Education plays a crucial role in physiotherapy. It involves clearly explaining the diagnosis and available treatment options to ensure that the patient fully understands their condition. Additionally, it is essential to identify and address any oral habits that may be potentially harmful.

In the context of education, providing guidance on the biomechanics of the jaw, neck, and head is important. This includes guidance on optimal posture and movement mechanics to mitigate preventable pain and musculoskeletal strain. Furthermore, comfort and emotional support play a significant role in helping patients throughout their treatment [6,11].

Cognitive-behavioral therapy (CBT) is also an essential component in the management of symptoms associated with TMD. It aims to modify catastrophic thoughts, behaviors, and maladaptive emotional responses associated with pain, thereby improving pain coping mechanisms and general well-being. Through cognitive restructuring, it helps patients modify catastrophic thoughts (“my pain is unmanageable”) and develop a more constructive perception of their condition. Relaxation techniques, such as deep breathing and progressive muscle relaxation, directly reduce tension in the jaw and surrounding muscles. CBT also breaks the vicious circle of fear-avoidance by gradually reintroducing normal chewing and mouth-opening activities, thus preventing the worsening of symptoms due to inactivity. At the same time, it improves stress and emotion management, key factors in chronic TMD, while enhancing patients' self-efficacy in the face of painful episodes [12].

Turner et al. conducted a randomized clinical trial to assess the impact of this approach on pain associated with chronic TMD. The results demonstrated that over 50% of participants in the experimental group experienced a reduction in pain intensity greater than 50%, compared to only 29% in the control group. Moreover, patients who underwent CBT also exhibited improvements in masticatory function and a decrease in depressive symptoms [13].

Physical therapy

Physical therapy includes the application of cold and heat, ultrasound, laser therapy, electromyography (EMG) coupled with biofeedback, and transcutaneous electrical nerve stimulation (TENS).

Cold

Cold, or cryotherapy, plays an essential role in the treatment of temporomandibular dysfunction, relieving pain and reducing muscle inflammation. It acts through several physiological mechanisms. Firstly, it induces vasoconstriction (reduction in blood vessel diameter), which reduces local blood flow, thus helping to reduce inflammation. Secondly, it reduces pain by slowing nerve conduction in sensory fibers, reducing nociceptive activity in skin tissue, while limiting the release of algogenic mediators. Thirdly, it induces muscle relaxation (myorelaxation), attenuating muscle spasm and rigidity. Fourthly, it inhibits inflammatory processes by reducing the cellular metabolism of inflammatory agents, reducing the frequency of enzymatic reactions, lowering inflammatory mediators, and, secondly, improving the vascularization of injured tissues [14,15].

Cryotherapy can be applied in different ways [15], such as using ice packs for 10 to 15 minutes, two to four times a day, on the masticatory muscles (especially the temporal and masseter muscles), on painful points, or directly on the TMJ. Another method involves steam-cooling sprays, applied for about 10 seconds, often before muscle-stretching exercises.

Heat

Superficial thermotherapy is an approach commonly used in the treatment of temporomandibular dysfunction, offering several therapeutic benefits. The application of heat induces vasodilation, which improves blood circulation and tissue oxygenation, while reducing pain-related nerve conduction and promoting the elimination of metabolites responsible for muscle pain. It also relaxes the masticatory muscles and increases mandibular mobility, with gains in mouth opening of up to 9 mm [16].

Application techniques vary between moist heat (hot towels, water/frost bags wrapped in a damp cloth) and dry heat (electric cushion or hot water bottle). Although moist heat is generally preferred for its better thermal conduction and patient acceptance, some studies show no significant difference in efficacy between the two methods. The recommended duration of application is 20 minutes per session, with a frequency of one to three times a day for acute cases. The ideal temperature is between 40°C and 42°C, a safe and effective range for TMD patients who often present increased sensitivity to heat [17].

EMG Coupled With Biofeedback

EMG, coupled with biofeedback, uses a skin sensor to record electromyographic activity. This sensor allows the patient to visualize, on a screen, the level of contraction in the muscle located beneath the electrode (such as the masseter or temporalis). By observing the screen, the patient receives feedback through both visual and audible signals. This process aims to increase the patient's awareness and control over the movements to be performed, helping them learn to regulate muscle activation and, consequently, better perceive the muscle's resting posture [6,9,10].

Transcutaneous Electrical Nerve Stimulation (TENS)

Then there is electrical nerve stimulation, mainly used to manage persistent pain and to relax the masticatory muscles.

The beneficial effects of TENS on TMDs can generally be explained by two theories. The first is that it stimulates the motor nerves, causing rhythmic contraction of the masticatory muscles, which increases blood flow and oxygen, thereby reducing edema and the accumulation of harmful toxins. This reduces pain and fatigue in the chewing muscles. According to the second theory, tactile and pressure stimulation inhibit structures that provide stimulation to sensory neurons by electrical means (gate theory) [9].

According to the systematic review by Serrano-Muñoz et al., the use of TENS resulted in a clinically significant reduction in pain in patients with TMDs. Protocols varied from study to study, with frequencies ranging from 0.5 to 100 Hz, pulse widths from 50 to 500 ms, and intensity adjusted to be well tolerated, with or without muscle contraction. Session durations ranged from 20 to 60 minutes, for a total of between one and 70 sessions. Electrodes were generally placed on the masseter and anterior temporal muscles, or directly in the region of the TMJ [18].

Ultrasound

Ultrasound uses high-frequency sound waves to generate heat deep within the tissue [1,10]. Khairnar et al. [19] conducted a comparative study in which they applied ultrasound at an intensity of 1.8 W/cm² for 10 minutes per session. They concluded that this modality is the treatment of choice for reducing pain and inflammation associated with TMJ disorders.

The application of ultrasound can cause local heating of tissues, which improves blood circulation and promotes healing. A slightly elevated temperature in the treatment area may reduce pain and inflammation in the TMJ region, stimulate cellular metabolism, and accelerate tissue repair [10].

Low-Level Laser Therapy (LLLT)

Laser therapy involves using red or near-infrared light to induce beneficial effects on cells or tissues. It can promote the release of endogenous opioids, improve tissue repair and cellular respiration, increase vasodilation and pain threshold, and reduce inflammation [20].

A combination of 660 nm indium gallium aluminum phosphide (InGaAlP) laser and 890 nm gallium arsenide (GaAs) laser applied to the masticatory muscles of patients with muscular TMDs, and a combination of 650 nm InGaAlP and 830 nm GaAs lasers applied to the TMJ area of patients with joint TMDs, resulted in effective pain reduction when applied twice a week for three weeks [21].

Manual therapy

Manual therapy encompasses a range of techniques aimed at restoring the normal range of motion, reducing local ischemia, stimulating proprioception, breaking down fibrous adhesions, stimulating synovial fluid production, and reducing pain [6,9,10,22-24].

Massage

Massage techniques used in therapeutic procedures include several types of movement, each with its own specific method of execution and results. Effleurage and kneading are massage techniques involving soothing, stroking, and circular movements applied to the skin and underlying tissues. These maneuvers are generally performed at the beginning or end of the session to warm up the muscles, stimulate blood and lymph circulation, and improve perfusion of the small vessels in the targeted tissues. For the masseter muscle, the therapist uses the tips of the thumb, index, and middle fingers to exert circular pressure on different points of the muscle. For the temporalis muscle, the therapist places the thumbs on the patient's forehead, the index and middle fingers at the temples, and the ring fingers behind the ears, then makes gentle circular movements with light pressure on the muscle tissue. Friction is another manual technique that involves applying targeted digital pressure to trigger points, gradually increasing the intensity until muscle relaxation is achieved. This method promotes local reconstruction of the muscle microstructure and relieves pain in a punctual manner by activating the pain control mechanism, according to the “gate control” theory. Finally, stretching (petrissage) involves rolling movements to lengthen muscle fibers. This technique increases joint amplitude, reduces excessive muscular contractions, and contributes to a more balanced joint [9,25].

Body Posture

In manual therapy, attention is also given to the posture of the body, particularly that of the head, as it affects the position of the jaw.

The postural techniques, integrated with a 12-month CBT, aim to correct poor daily postural habits to reduce muscle tension and improve mandibular function. They include advice on how to sit, stand, sleep, walk, and eat properly, as well as how to avoid harmful gestures such as crossing the legs or throwing the head forward [26]. In addition, a number of specific exercises are prescribed: the "chin tuck" to realign the head, the "chest stretch" to open the rib cage, the "wall stretch" to correct back alignment, the "lengthened stretch" to relax the shoulders, and "flat stomach arm lifts" to strengthen the upper back. Together, these techniques promote balanced posture, relieve pain, improve mouth opening, and prevent relapses in patients suffering from myogenic TMD [27].

Therapeutic Manipulations and Exercises

Joint mobilizations: These techniques help improve the range of motion, reduce pain, and alleviate muscle spasms. They involve gently moving the mandible in specific directions, such as propulsion, vertical distraction, and lateralization-medialization [6].

Therapeutic exercises: These exercises include stretching, counter-resistance, and strengthening. They help improve muscle coordination, relax tense muscles, increase the range of motion, and work on proprioception and muscular endurance [9].

Stretching exercises: The purpose of stretching is to restore the efficiency of lubrication and stimulate the synthesis of glycol-aminoglycan between collagen fibers, which allows movement in the peri-articular structures. Stretching exercises are beneficial for both arthrogenous and myogenous TMDs [28]. These exercises include lateral jaw movement and opening stretches. For lateral movement, the exercise begins with gentle pressure on the mandibular body to gradually increase amplitude, followed by a release phase. This pressure-release cycle is repeated twice, then on the third repetition, maximum pressure is applied and maintained for 15 seconds. Then, for the opening stretches, the practitioner uses their thumbs to create an opening between the thumb and index finger. At home, the patient opens the mouth to its maximum spontaneous limit, then applies three gentle downward pressures with two fingers placed on the lower incisors, releasing between each pressure, while holding the third for 15 seconds [3].

Counter-resistance exercises: The patient opens the mouth to the first level of muscular tension, then exerts a slight contraction without movement against the practitioner’s resistance. The counter-resistance exercise targets the depressor muscles during mouth opening and the elevator muscles during mouth closing [3].

Mobilization/Cervical Manipulation

There is a close biomechanical relationship between the upper cervical spine and the TMJ, so cervical mobilization techniques, particularly in segments C0 to C3, have been shown to have a beneficial effect on mandibular function. By restoring the synergy between cervical movements and masticatory muscle activity, these manipulations correct the joint dysfunctions responsible for the mouth-opening limitations frequently observed in TMDs. Clinical results confirm the value of these manual approaches in simultaneously improving cervical mobility, mandibular amplitude, and pain control [24,29].

In patients with TMD, cervical flexion mobilization helps to correct the kyphosis often present in the C3-C4 vertebrae, while posterior-anterior central mobilization, targeting the C4-C5 levels, specifically targets C4 to treat the deformities observed. In addition, craniocervical flexor stabilization exercises enhance the electromyographic activity of the deep neck muscles, essential for cervical spine stability. Maintained apophyseal gliding, used in the treatment of cervical headaches, significantly improves cervical flexion, while stretching exercises targeting the cervical muscles are indicated to correct head and neck misalignments linked to muscle shortening. The systematic integration of these manual and functional interventions is therefore essential in the overall management of TMD [28].

Self-rehabilitation

Self-rehabilitation includes all exercises performed independently by the patient, based on the practitioner’s instructions. These exercises may be regularly supervised by a physiotherapist and encompass self-massage and techniques aimed at correcting a deflected jaw opening.

Self-Massage

For the masseter muscle, the patient identifies the painful area and muscle mass by applying pressure for at least one minute. This maneuver is performed by placing the thumb intraorally and two fingers extra orally. Then, the muscle is stretched by applying pressure with the thumb, moving from its origin at the zygomatic arch to its insertion at the angle of the mandible.

The temporalis muscle is massaged by making circular movements with the palm of the hand or fingers, or by pressing on the painful area for 10 seconds [3].

Correction of Deflected Oral Opening

This exercise involves slowly opening and closing the mouth in front of a mirror, ensuring that there is no deviation of the jaw during the movement. The same movement should then be repeated with the hands placed on the TMJs to better control the motion. The exercise should be performed three times a day, with 20 repetitions per session [3].

## Conclusions

Like other musculoskeletal disorders, TMDs can be effectively managed using the various therapeutic modalities mentioned above. These disorders significantly impact the quality of life of those affected due to the chronic pain and functional limitations they cause. Existing research indicates that no single therapeutic approach alone provides substantial and lasting symptom relief. On the other hand, multidisciplinary care, combining medical, physical, behavioral, and sometimes psychological interventions, proves essential in promoting complete remission and improving the overall well-being of patients.
